# A Systematic Study on Bio-Based Hybrid Aerogels Made of Tannin and Silica

**DOI:** 10.3390/ma14185231

**Published:** 2021-09-11

**Authors:** Ann-Kathrin Koopmann, Wim J. Malfait, Thomas Sepperer, Nicola Huesing

**Affiliations:** 1 Department of Chemistry and Physics of Materials, Paris-Lodron University of Salzburg, 5020 Salzburg, Austria; ann-kathrin.koopmann@sbg.ac.at; 2 Salzburg Center for Smart Materials, 5020 Salzburg, Austria; thomas.sepperer@fh-salzburg.ac.at; 3 Swiss Federal Laboratories for Materials Science and Technology, Empa, 8600 Dübendorf, Switzerland; wim.malfait@empa.ch; 4 Forest Products Technology and Timber Constructions, Salzburg University of Applied Sciences, 5431 Kuchl, Austria

**Keywords:** porous materials, aerogels, tannin, silica

## Abstract

Tannin-silica hybrid materials are expected to feature excellent mechanic-chemical stability, large surface areas, high porosity and possess, after carbothermal reduction, high thermal stability as well as high thermal conductivity. Typically, a commercially available tetraethoxysilane is used, but in this study, a more sustainable route was developed by using a glycol-based silica precursor, tetrakis(2-hydroxyethyl)orthosilicate (EGMS), which is highly water-soluble. In order to produce highly porous, homogeneous hybrid tannin-silica aerogels in a one-pot approach, a suitable crosslinker has to be used. It was found that an aldehyde-functionalized silane (triethoxysilylbutyraldehyde) enables the covalent bonding of tannin and silica. Solely by altering the processing parameters, distinctly different tannin-silica hybrid material properties could be achieved. In particular, the amount of crosslinker is a significant factor with respect to altering the materials’ properties, e.g., the specific surface area. Notably, 5 wt% of crosslinker presents an optimal percentage to obtain a sustainable tannin-silica hybrid system with high specific surface areas of roughly 800–900 m^2^ g^−1^ as well as a high mesopore volume. The synthesized tannin-silica hybrid aerogels permit the usage as green precursor for silicon carbide materials.

## 1. Introduction

Highly porous materials, particularly aerogels, became of great interest during the last decades since their first production in the 1930s by Kistler via supercritical fluid extraction. Aerogels are characterized by a combination of remarkable structural properties [1]: they are low-density (usually <0.2 g cm^−3^), nanoporous solids with large surface areas (500–1000 m^2^ g^−1^) combined with a high porosity (>90% *v*/*v*), typically obtained by supercritical drying of a wet gel, preserving the volume and, hence, the pore structure of the gel [2,3,4].

According to their chemistry, aerogels can be classified either as inorganic, organic or as hybrid of both. Inorganic aerogels, in particular silica aerogels, are known to have extremely low thermal conductivities and high surface areas. Organic aerogels, in contrast, have, in general slightly, lower surface areas and higher thermal conductivities; however, they feature distinctly better mechanical properties and can be easily converted to the corresponding carbon aerogels [5,6].

Traditionally, the three-dimensional network formation of silica gels is accomplished by hydrolysis and condensation of alkoxysilanes. Typically, tetraethylorthosilicate (TEOS) or tetramethylorthosilicate (TMOS) have been used, which, however, require the presence of a co-solvent, typically an alcohol [7,8]. In contrast to that, the utilization of a glycol-based silica precursor namely tetrakis(2-hydroxyethyl)orthosilicate (EGMS), as proposed by Huesing et al. [9,10,11], is beneficial as it is water-soluble and, hence, can easily be hydrolyzed/condensed under neutral and aqueous conditions without any co-solvent. Furthermore, it was recently shown that EGMS can easily be obtained from biogenic, sustainable sources, i.e., silica from rice hull ash [12].

The most intensively studied precursor for organic aerogels is resorcinol-formaldehyde (RF), as first described by Pekala in 1989 [13]. Polyurethane, polyimide and polyurea based aerogels describe other frequently studied organic aerogel systems. In addition, biopolymer-derived aerogels, mostly based on polysaccharides, provoke increased interest due to their sustainable character [5,14]. However, in terms of sustainability, considerable research has been performed focusing on the prevalent RF organic aerogel system. More precisely, the precursors resorcinol and formaldehyde imply high costs as well as high toxicity; hence, alternatives for novel, cheaper, non-toxic and eco-friendlier materials that show a similar reactivity as resorcinol and formaldehyde, have been searched for [15]. In order to exchange resorcinol with a greener alternative, several low-cost phenolic compounds such as phenol itself, cresol, tannin and lignin have been studied [15]. In particular, the usage of the polyphenolic tannin, which is a natural, sustainable and low cost material (roughly 1.50 EUR kg^−1^), has been reported extensively allowing the preparation of highly porous tannin-formaldehyde (TF) gels [15,16]. Overall, it was found that condensed tannins are highly suitable for the generation of homogeneous aerogels either in the presence of a crosslinker, such as formaldehyde, or by mixing it with other synthetic or natural molecules [17]. Kraiwattanawong et al. were the first to report that condensed tannins can easily be crosslinked and function as carbon precursor material [18,19,20]. Based on this, the group of Celzard and Pizzi has done extensive research on TF gels in order to reduce the overall production costs for carbon gels [16]. The polymerization and crosslinking of tannin and formaldehyde was found to be similar to the one of resorcinol and formaldehyde, as the flavonoid units of the tannin are linked to the aldehyde via mainly methylene and occasionally via methylene-ether bridges [17]. Therefore, the resulting TF gels show comparable properties to RF gels and, hence, TF aerogels are also suitable for similar applications.

Recently, composite (hybrid) materials/aerogels have provoked increased interest as they combine the favorable previously stated properties of both organic and inorganic precursors and, furthermore, might even yield novel characteristics that neither of the components on its own possess [2,4,21]. The generation of a hybrid material by combining an inorganic precursor (e.g., silica) with an appropriate organic one could benefit the mechanical properties of the resultant material by preventing the shrinkage and cracking upon the drying step, which is a common problem for porous inorganic materials [22]. Tannin-silica hybrid materials in form of multilayer particles or films and their applicability as, e.g., metal adsorbents or stimuli-responsive drug delivery systems, have been demonstrated in literature [23]. The generation of these hybrid materials often involves a pre-functionalization step of the ceramic materials, such as amination [24,25]. However, only a few studies on hybrid gels made up of a polyphenolic organic source as well as silica as inorganic source can be found in literature. Almost entirely, studies have investigated the utilization of the phenolic lignin and SiO_2_ for biocomposite hybrid materials. However, these studies predominantly focus rather on the preparation of hybrid xerogels and precipitates [21,26,27]. To the best of our knowledge, there are no reports concerning the generation of light-weight, highly porous, sustainable biocomposite aerogels, consisting of a green polyphenolic tree extract as well as silica. However, such kind of material could be profitable to have, as it represents a sustainable precursor for, e.g., silicon(oxy) carbide (Si(O)C) materials [28]. These SiC materials are of significant relevance due to their excellent physical and chemical properties, such as high thermal conductivity, superior chemical inertness, good thermal shock resistance and high mechanical stability, as well as their wide scope of application, i.e., electrode material, catalyst support and absorbent [29,30,31,32]. Hence, carbothermally treated polyphenolic-silica hybrid aerogels could illustrate a bio-based alternative to SiC materials, which are made of resorcinol-formaldehyde, as well as a silica precursor, whereby mostly water-insoluble TEOS or TMOS are used [29,30].

This study presents a one-pot approach for the generation of tannin-silica hybrid aerogels from sustainable precursors in an aqueous medium. A systematic characterization on their synthesis-structure relationship is presented, as well as their suitability as bio-based precursor for silicon carbide materials.

## 2. Materials and Methods

### 2.1. Chemicals

Technical grade ethanol (96%) and sodium hydroxide pellets were provided by VWR. As the source of condensed flavonoid tannin, a commercially available Mimosa extract (Weibull AQ) from the company Tanac (Montenegro, Brazil) was used. Hydrochloric acid (37%) was supplied by Merck. Triethoxysilylbutyraldehyde was purchased from Gelest. Tetrakis(2-hydroxyethyl)orthosilicate (EGMS) was synthesized according to the method published by Huesing and coworkers via a transesterification process of tetraethylorthosilicate and ethylene glycol [10,11].

### 2.2. Synthesis of Tannin-Silica (TS) Hybrid Aerogels

Tannin-silica hybrid gels with a theoretical density of 0.1 g cm^−3^ were prepared accordingly: Mimosa tannin was mixed with deionized water. The water-insoluble tannin fraction (approximately 4 wt%) was centrifuged off (4500 rpm for 30 min) and discarded. Afterwards, the pH of the remaining solution was adjusted to either pH 3 or pH 6 using 1 M HCl or 0.1 M NaOH. Then, EGMS was added to the solution. The sol was stirred for five minutes and filled in polypropylene (PP) screw-capped cylindrical tubes (h: 5.3 cm, w: 1.3 cm). These tubes were then stored vertically at 80 °C in order to promote gelation of the sol and aging of the gels. After seven days, the monolithic hydrogels were liberated from the PP tubes. The gels were washed at least five times with ethanol (roughly 50 mL) prior to drying by supercritical extraction with CO_2_ (60 °C, 110 bar) [33]. In order to investigate a wide range of TS gels, the pH value and the tannin/silica (T/S) weight ratio was varied while keeping the theoretical density of the batches constant. For better crosslinking and thus, more stable three-dimensional gel networks, the addition of variable amounts of triethoxysilylbutyraldehyde, an aldehyde-functionalized silane crosslinker, was studied. Therefore, the crosslinker, with mass percentages of either 2, 5 or 10% in regard to the total amount of the sol, was added immediately after addition of EGMS. The generated tannin-silica hybrid aerogels were labelled as TS_a_b_c, where a defines the nominal tannin/silica mass ratio, b the initial pH value of the sol and c the percentage of amount of crosslinker added (Figure S1). The exact amounts used for the different batches are displayed in the supporting information (Table S1). 

In order to carbonize the tannin precursor as well as reduce silicon, dried TS aerogels were carbothermally reduced in a Nabertherm tube furnace at 1500 °C under argon atmosphere (75 L h^−1^) for 5 h with a heating rate of 5 °C min^−1^.

### 2.3. Characterization of Hybrid Tannin-Silica Aerogels

The bulk density ρ_b_ (g cm^−3^) is defined as the material’s mass divided by the total volume it occupies. The bulk density was determined by weighing a sample of known dimensions and dividing it by the volume of the cylindrical sample according to the following formula, whereby m corresponds to the mass, r to the radius and h to the height of the cylindrical monolith.

ρ_b_ = m/r^2^πh

The skeletal density ρ_s_ (g cm^−3^) was obtained by analyzing small pieces of the monoliths using helium pycnometry, which was performed on a ULTRAPYC 1200 e automatic density analyzer Quantachrome instrument. Determination of the bulk and skeletal density allows the calculation of the overall porosity Φ of the sample according to the following formula:

Φ = 1 − (ρ_b_/ρ_s_)

The diametric shrinkages of the gels after supercritical drying were recorded by measuring the samples’ diameter after supercritical drying and comparing the gained diameter with the inner diameter of the PP containers.

In order to determine the pore texture of the aerogels, nitrogen adsorption analysis was carried out on a Sy-Lab Micromeritics ASAP 2420 surface area and porosity analyzer. The adsorption and desorption isotherms were recorded at −196 °C in a relative pressure range *p*/*p*_0_ from 10^−7^ to 1 after degassing the sample prior analysis at 80 °C for 24 h under vacuum. Analysis of the gained isotherms was carried out using the MicroActive (Version 5.0) software. The specific surface area S_BET_ was calculated using the Brunauer, Emmett and Teller method [34]. The mesopore volume was determined from the adsorption branch by using the DFT model, assuming slit pore geometry and considering pore sizes of up to 40 nm. Furthermore, the pore size distribution for the nitrogen isotherm’s desorption branch has been analyzed by using the Barret, Joyner and Halenda method [35].

The synthesized tannin-silica hybrid aerogels were analyzed regarding their behavior upon heating. The weight loss of the sample as a function of temperature was determined by thermogravimetric analysis (TGA) using a Netzsch STA 449 F3 Jupiter instrument (Selb, Germany). The sample was heated at a rate of 10 °C min^−1^ to 1000 °C in air. Transmission electron microscope (TEM) images were recorded with JEOL JEM F200 TEM (Akishima, Japan), which is equipped with a cold field emission source and uses a TVIPS F216 2k by 2k CMOS (Gauting, Germany) camera. Moreover, scanning transmission electron microscopy (STEM) images, displaying the z-contrast as well as EDX intensity maps were obtained in STEM mode with a typical beam current of 0.1 nA and a beam diameter of 0.16 nm during 15 min. The maps were obtained by signal integration of counts over Si Kα transition line for Si (integration: 1.63–1.89 keV), O Kα line for O (integration: 0.46–0.59 keV) and C Kα line for C (integration: 0.21–0.34 keV). 

The degree of crystallinity of the thermally treated tannin-silica aerogels were analyzed by X-ray diffraction, which was performed on a Bruker D8 Advance instrument, using Cu-Kα radiation (40 kV, 40.0 mA). The composition of the TS material, after carbothermal reduction, was investigated using the diffractometer software EVA V5.0 and quantified by employing the TOPAS software. Raman spectra of the carbonized tannin-silica aerogels were recorded by a DXR2 Raman Microscope (Thermo Scientific; Waltham, MA, USA) using a 532 nm laser excitation wavelength and a laser power of 4 mW.

Solid-state NMR spectra were acquired with a Bruker Avance III spectrometer equipped with a 9.4 T wide-bore magnet, corresponding to Larmor ratios of 400.2, 100.6 and 79.5 MHz for ^1^H, ^13^C and ^29^Si, respectively. The spectra are referenced to tetramethylsilane, using adamantane (^13^C) and silicone rubber (^29^Si) as secondary, external chemical shift standards. All spectra were acquired with 7 mm zirconia rotors under magic angle spinning (MAS) at a rate of 4000 Hz. ^1^H-^13^C and ^1^H-^29^Si spectra were collected with cross polarization (CP) to maximize sensitivity. A repetition rate of 3 s was employed with 866–2995 and 8952–34,739 acquisitions for the ^1^H-^13^C and ^1^H-^29^Si CP spectra, respectively. Relatively long contact times (2 ms for ^1^H-^13^C and 5 ms for ^1^H-^29^Si CP) were selected to reduce the effect of ^1^H-X distance on the relative spectral intensities. The samples’ weights were recorded to enable normalization and cross-sample comparison.

## 3. Results and Discussion

### 3.1. Formation of Tannin-Silica Networks

In a first set of experiments, the formation of a three-dimensional hybrid tannin-silica gel network by just mixing tannin and EGMS without any further crosslinker was evaluated. Two different pH values, i.e., 3 and 6, were tested to probe the effect of the different surface charges on the chemical and particle interactions. The gel’s network formation is on the one hand, particularly influenced by the protonation or deprotonation of the tannins’ hydroxyl groups in acidic or alkaline media, respectively. Moreover, under acidic conditions the degradation of the tannins’ polymeric chain and condensation of the flavonoid units as well as under alkaline conditions the epimerization and rearrangement of the tannins C-ring have to be considered [36]. On the other hand, the hydrolysis and condensation of EGMS is significantly affected by the pH. Under acidic conditions, the hydrolysis is favored and the condensation is the rate-determining step, whereas under alkaline conditions the reaction rates of hydrolysis and condensation are reversed [37]. Overall, the aim was to produce covalent tannin-silica networks via the formation of Si-O-C connections, however, keeping in mind that an alkoxide-based connection can only provide limited stability against hydrolysis (Supporting Information, Figure S2 and Table S2, photographs of gels and their characterization). Thermogravimetric analysis (TGA) supported the hypothesis that this bonding yields unstable connections, as under the afore-mentioned conditions nearly exclusively pure silica networks are produced with only minor traces of tannin (the calculation of amount of tannin present in the network and representative TG curves is given in the Supporting Information, Page S5 and Figure S3). Overall, only minor fractions of tannin (11–4%) are retained in the network during the solvent exchange or the drying procedure. Presumably, these minor tannin fractions are blocked within the pores of the silica networks since an increase of the T/S ratio (0.15 to 1.0), hence, decreasing the amount of silica compared to the amount of tannin, yields a lower amount of retained tannin indicating less tannin blocked in pores.

**Figure 1:**
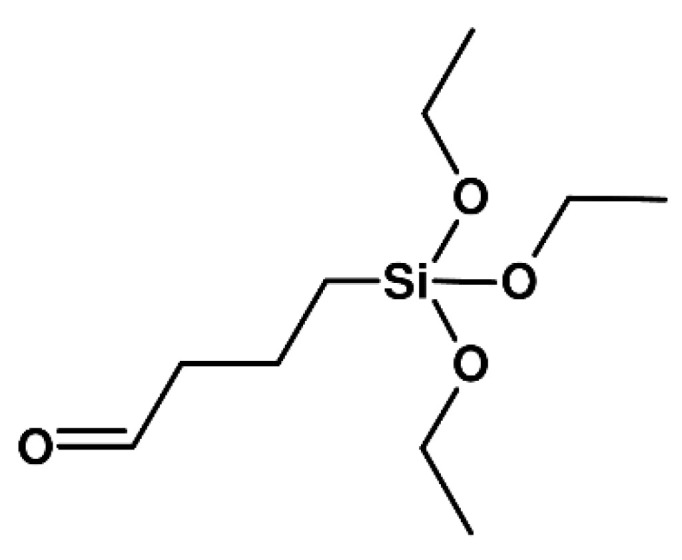
Structural formula of triethoxysilylbutyraldehyde (4-triethoxysilylbutanal).

Hence, for the synthesis of a true hybrid network of tannin and silica, the addition of a crosslinker is required. Desired characteristics of a suitable crosslinker are the ability to covalently crosslink to the tannin molecule via, i.e., the aid of an aldehyde moiety as well as the capability to form siloxane bridges to the silica backbone. Therefore, an aldehyde-functionalized silane (AS), namely triethoxysilylbutyraldehyde, has been selected (Figure 1) and the proposed bonding situation is depicted in Figure S4. In the scope of a systematic study for crosslinked tannin-silica gels with a theoretical density of 0.1 g cm^−3^, several process parameters were varied, namely the pH values (3 and 6), the T/S ratio (0.15, 0.5 and 1.0), and amount of the crosslinker (2, 5 and 10 wt%). Photographs of the resultant gels are visualized in Figure S2.

**Figure 2:**
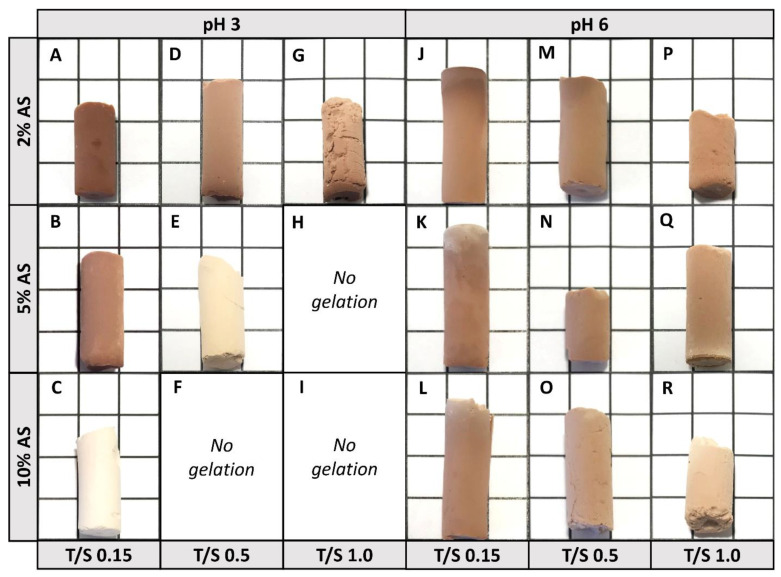
Photographs of the hybrid gels, illustrated on a 1 cm^2^ grid, TS_0_._15_3_2_ (**A**), TS_0_._15_3_5_ (**B**), TS_0_._15_3_10_ (**C**), TS_0_._5_3_2_ (**D**), TS_0_._5_3_5_ (**E**), TS_0_._5_3_10_ (**F**), TS_1_._0_3_2_ (**G**), TS_1_._0_3_5_ (**H**), TS_1_._0_3_10_ (**I**), TS_0_._15_6_2_ (**J**), TS_0_._15_6_5_ (**K**), TS_0_._15_6_10_ (**L**), TS_0_._5_6_2_ (**M**), TS_0_._5_6_5_ (**N**), TS_0_._5_6_10_ (**O**), TS_1_._0_6_2_ (**P**), TS_1_._0_6_5_ (**Q**) and TS_1_._0_6_10_ (**R**).

Most of the investigated formulations allow the generation of a three-dimensional gel network except for the batches TS_0_._5_3_10_; TS_1_._0_3_5_ and TS_1_._0_3_10_, where no gelation could be observed. For all others, gels were obtained with gel times that varied strongly between the different batches (Supporting Information, Table S3). In general, a higher pH value, i.e., close to neutral conditions, results in distinctly faster gelation (within several minutes) compared to the lower pH (gels within hours), whereby the gelation time is even faster for lower T/S ratios. Moreover, the amount of added crosslinker has an impact on the gelation time since the more crosslinker added, the slower the gelation takes place. Additionally, differences in color are observed (Figure 2) and these variations cannot be solely explained by the different amounts of tannin retained. Likely, the color is also affected by differences in the particle size that form the three-dimensional network. As known from literature, the particle size is increasing with an increasing pH value for the tannin-based aerogel system, ranging from a few to tens of nm in diameter [38]. Due to variations in the nanoparticles’ diameter, the resultant three-dimensional network differs in its morphology leading to differences in their light absorption and, hence, in their visual appearance.

**Figure 3:**
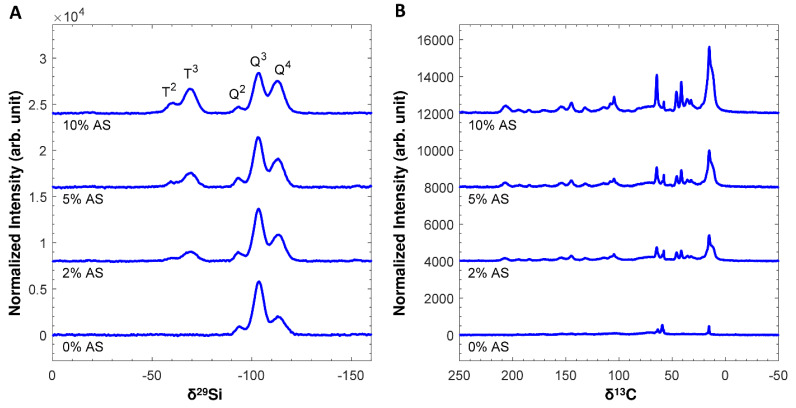
^1^H-^29^Si CP MAS NMR spectra (**A**) and ^1^H-^13^C CP MAS NMR spectra (**B**). All spectra are normalized to the same sample mass and same number of scans. The assignment of the ^29^Si resonances is indicated in the figure. Please refer to Figure S5 and Table S4 for the peak assignment of the ^13^C spectra.

First, in regard to the tannin-silica network formation, the chemical structure and composition of the resultant gel network is investigated. For simplicity reasons the TS series with a T/S ratio of 0.15, a pH value of 6 as well as various amounts of crosslinker (see Figure 2J–L) has been selected. Solid state NMR spectroscopy has been performed to obtain a better understanding of the chemical bonding situation within the hybrid network. Figure 3 displays the ^1^H-^29^Si and ^1^H-^13^C CP MAS NMR spectra of tannin-silica hybrid aerogels, prepared with increasing amounts of triethoxysilylbutyraldehyde (2 to 10 wt%). Please note that these CP spectra are not inherently quantitative. Spectral intensities do scale with species concentration but are also influenced by variations in cross polarization and relaxation efficiencies. Nevertheless, the peak intensities provide a qualitative measure of sample composition and because the spectra are normalized to the same number of scans and sample mass, peak intensities can be compared across different samples.

**Figure 4:**
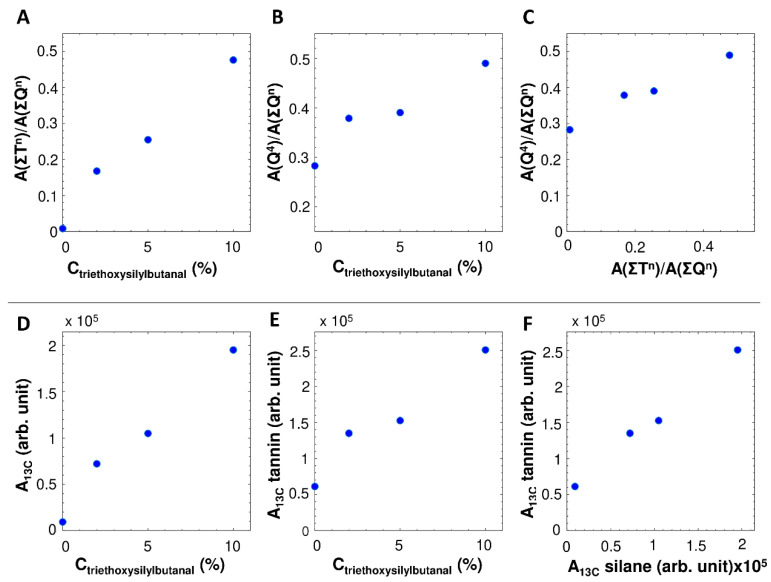
Integrated ^1^H-^29^Si CP MAS NMR (**A**–**C**) and ^1^H-^13^C CP MAS NMR (**D**–**F**) peak areas. (**A**) Increase in T^n^ peak area (−50 to −80 ppm) with increasing trialkoxysilane concentration. (**B**) Increase in silica polymerization (Q^4^ peak area, see Figure S6) with increasing trialkoxysilane concentration. (**C**) Increase in silica polymerization with increasing T^n^ peak area. (**D**) Increase in intensity of the silane peak area (5 to 22 ppm) with increasing trialkoxysilane concentration. (**E**) Increase in tannin peak area (60 to 162 ppm) with increasing trialkoxysilane concentration. (**F**) Correlation between the tannin and silane peak intensities.

The ^1^H-^29^Si CP MAS NMR spectra display the bands expected for a silica aerogel functionalized with a trialkoxysilane (Figure 3A) [39,40]: (i) two bands near −60 and −68 ppm related to T^2^ and T^3^, respectively, where T^n^ is a Si atom bonded to three oxygen atoms (n bridging oxygen and 3-n non-bridging oxygen atoms) and one carbon atom related to the crosslinker; and (ii) three bands near −93, −103 and −112 ppm related to Q^2^, Q^3^ and Q^4^, respectively, where Q^n^ is a Si atom coordinated by n bridging oxygen and 4-n non-bridging oxygen atoms, derived from the hydrolysis-condensation of EGMS. For all samples, the degree of polymerization is rather high, with no visible peaks for T^0^, T^1^, Q^0^ and Q^1^ and the highest peak intensities for T^3^ and Q^3^. As expected, the T^n^ band intensities scale with the amount of triethoxysilylbutyraldehyde (Figure 4A), confirming the increased incorporation of the trialkoxysilane in the aerogels. In addition, the degree of polymerization, exemplified by the intensity of the Q^4^ band, increases for increasing amounts of triethoxysilyl butyraldehyde (Figure 4B) or increasing T^n^ intensity (Figure 4C). This confirms the grafting of trialkoxysilanes on the silica surfaces, through Q-O-T condensation reactions, e.g., Q^3^-OH + T^2^-OR = Q^4^-O-T^3^ + ROH. Hence, at least a significant fraction of the triethoxysilylbutyraldehyde is present on the silica surfaces throughout the samples, rather than as separate phases or particles.

The ^1^H-^13^C CP MAS NMR spectra (Figure 3B) display the resonances expected for tannin [41], triethoxysilylbutyraldehyde and its reaction products (Supporting Information, Figure S5 and Table S4). The bands near 207 ppm and 46 ppm might correspond to unreacted butyraldehyde (C=O and neighboring CH_2_, respectively). However, another possible explanation for the band near 207 ppm could be due to the autocondensation of tannins associated with dimerization via ring opening and catechinic acid rearrangement, as proposed by Boehm et al. [42]. The peak near 36 ppm can be assigned to reacted butyraldehyde or can account, in case of the autocondensation of tannins, for the aliphatic carbon, generated via the ring opening mechanism. However, presumably, both unreacted and reacted butyraldehyde groups are present in the aerogels. The composite peak around 15 ppm can be assigned to the carbons of the trialkoxysilanes alkane chain closest to the Si atom. Perhaps the most important observation is the strong correlation between the tannin peak intensities with the triethoxysilylbutyraldehyde concentration (Figure 4E) and peak intensities (Figure 4F). This confirms the critical role of triethoxysilylbutyraldehyde as a covalent crosslinker to retain significant fractions of tannin in the final aerogel. Based on the obtained NMR data, no conclusive statement can be made concerning the structure of the hybrid gels, since the presence of unreacted butyraldehyde groups as well as the tannins’ autocondensation have to be taken into account. However, the autocondensation is more prevalent in procyanidins and prodelphinidins rather than in profisetidins and prorobinetinidins [43], whereas the latter one is the main extract of the used mimosa tannin. Hence, it is more likely that unreacted butyraldehyde is present in the network rather than an autocondensation of tannin took place. Overall, the NMR data suggest the presence of a silica macromolecular structure, with interconnected tannin to a certain extent, whereby the crosslinker benefits the retention of the tannin.

**Figure 5:**
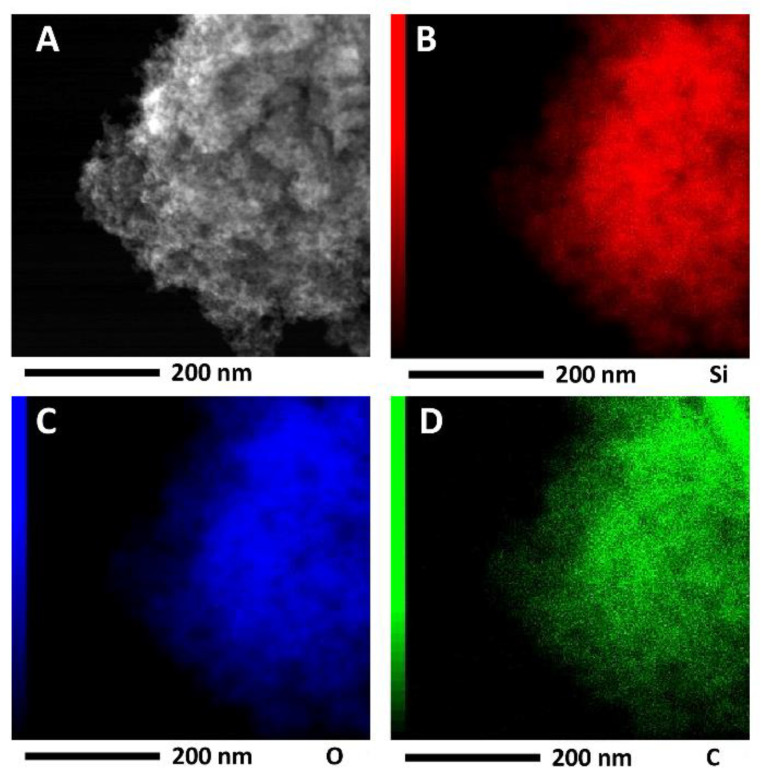
Transmission electron micrograph of the tannin silica aerogel (TS_0_._15_6_10_) (**A**) and the distribution of silicon (red, (**B**)), oxygen (blue, (**C**)) and carbon (green, (**D**)) atoms throughout the sample, visualized by STEM images.

Scanning transmission electron microscopy (STEM) affirms the incorporation of tannins inside the silica matrix, as already suggested by solid state NMR analysis, in a homogeneous manner (Figure 5). In detail, the red parts represent the silica matrix and the green parts the carbon areas, arising from the tannins and crosslinker.

**Figure 6:**
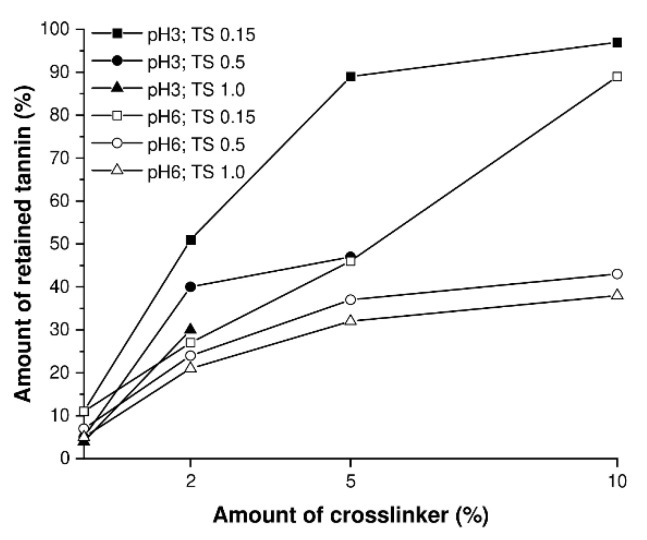
Graphic illustration of the amount of tannin retained in the tannin-silica network, which were generated at a pH value of 3 (full symbols) and 6 (empty symbol) as well as at different T/S ratios of 0.15 (square), 0.5 (hexagon) and 1.0 (triangle). Please note that the lines connecting the measured data points do not rely on a theoretical function but rather are used for clarity reasons.

The critical role of the crosslinker on the retention of the tannin, suggested by NMR analysis, is further substantiated by determining the amount of tannin retained in the silica network using TG analysis. Please note that the amount of retained tannin is only an estimation (details to the calculation made are found in the Supporting Information, page S5) and the exact amounts of tannin retained can negligibly differ, depending on the actual bonding situation. Analyzing the tannins’ retention of the above-named TS series (T/S ratio 0.15, pH 6), which is graphically illustrated in Figure 6 with empty squares, yields the same tendency as the NMR results (Figure 4E), since an increasing amount of crosslinker benefits the retention of the tannin. More precisely, for these tannin-silica networks, amounts of 0, 2, 5 and 10 wt% crosslinker account for 11, 27, 46 and 89% retained tannin, respectively. This behavior is determined as well for the other T/S ratios as well as for the pH value 3 (Figure 6 and Table S5 in Supporting Information). Furthermore, differences in the amount of retained tannin can be observed between the investigated T/S ratios. In particular, the T/S ratio of 0.15 has to be stressed as it depicts the largest deviation of retained tannin by varying the amount of crosslinker used. In contrast to that, only smaller deviations could be monitored for the batches with the T/S ratio of 0.5 and 1.0. Hence, supplementary to a high amount of used crosslinker, an initial excess of silica compared to tannin is needed for the generation of a tannin-silica hybrid system, in which most of the tannin and silica are covalently crosslinked.

### 3.2. Physical and Chemical Properties of Hybrid Tannin-Silica Aerogels

Based on the findings above, it can be concluded that the usage of triethoxysilylbutyraldehyde favors the generation of a homogeneously, covalently connected tannin-silica network. Furthermore, by simply altering the process parameters, i.e., the T/S ratio, the amount of crosslinker used as well as the adjusted pH value, the hybrid network formation of silica and tannin can essentially be adjusted since the amount of retained tannin varies widely between the different gel formulations. Therefore, it is assumed that the produced tannin-silica aerogels differ in their materials’ properties, such as porosity, mesopore volume and specific surface area (S_BET_) and, hence, presumably, also deviate in their applicability as commonly materials with low densities, high porosities and high surface areas are desired. First, as already assumed by the images of the dried gels (Figure 2), the different gels feature different degrees of diametric shrinkage depending on their processing pH value. Thus, the shrinkage as well as the porosity and density values have been determined and are illustrated in Table 1.

The TS aerogels, which have been prepared at a lower pH, show shrinkage values between 6–12% concomitantly with higher bulk densities of 0.09–0.18 g cm^−3^. In contrast to that, the TS hybrid gels prepared at a higher pH feature overall lower shrinkage values ranging from 1–6%. Thus, altering the pH by otherwise using the same formulation and drying procedure, allows the generation of a more stable aerogel structure, which withstands the supercritical drying process without noteworthy network structure changes. These overall lower shrinkages of the batches prepared at a higher pH also result in materials with lower densities (0.07–0.10 g cm^−3^) close to the theoretical density of 0.1 g cm^−3^. Deviations of a lower actual to a higher theoretical density have arisen from the extraction of tannin during the solvent exchange or drying step. Due to the comparably lower bulk densities of the gels prepared at a higher pH, they also feature generally higher porosity values (94–96%).

To further characterize the tannin-silica hybrid gels, nitrogen adsorption analysis was carried out, allowing the determination of the mesopore volume and specific surface area (S_BET_), which is defined as the total surfaces area of a material per unit of mass and is predominately a function of porosity, pore size distribution, shape, size and roughness (Table 1) [44].

**Figure 7:**
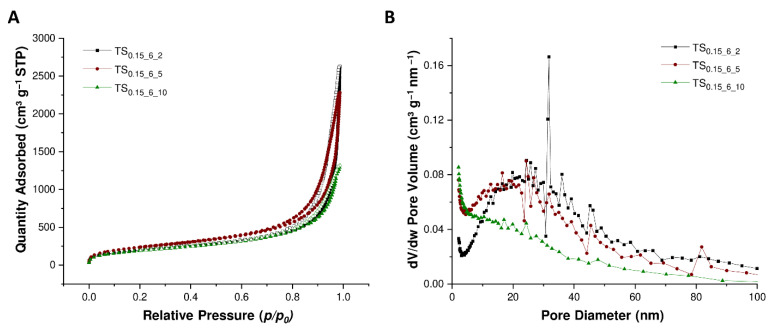
Nitrogen adsorption (full symbols) and desorption (empty symbols) isotherms (**A**) and the pore size distribution (**B**) of TS_0_._15_6_2_ (black), TS_0_._15_6_5_ (green) and TS_0_._15_6_10_ (red).

Representative for the tannin-silica aerogels, the nitrogen isotherms of the batches TS_0_._15_6_2_, TS_0_._15_6_5_ and TS_0_._15_6_10_ are presented in Figure 7. According to the IUPAC classification [45], all nitrogen isotherms of the tannin-silica aerogels are of type IV indicating a mesoporous structure. More precise, the type IV physisorption isotherm features the characteristic hysteresis loop, which is caused by capillary condensation processes taking place inside the mesopores as well as limited N_2_ uptake at high *p*/*p*_0_ values. Furthermore, the hysteresis loop is of type H3, as it shows no limiting adsorption at high *p*/*p*_0_ values, implying that plate-like particles with slit-shaped pores are present in the material [45]. Moreover, no or few micropores can be observed from the illustrated nitrogen isotherms. Since the isotherms do not level off completely at high relative pressure (*p*/*p*_0_) values, macropores are present as well in the resultant hybrid material. The specific surface area of the tannin-silica aerogels is ranging between 385 to 952 m^2^ g^−1^ and the mesopore volume accounts 0.2 to 2.0 g cm^−3^.

**Figure 8:**
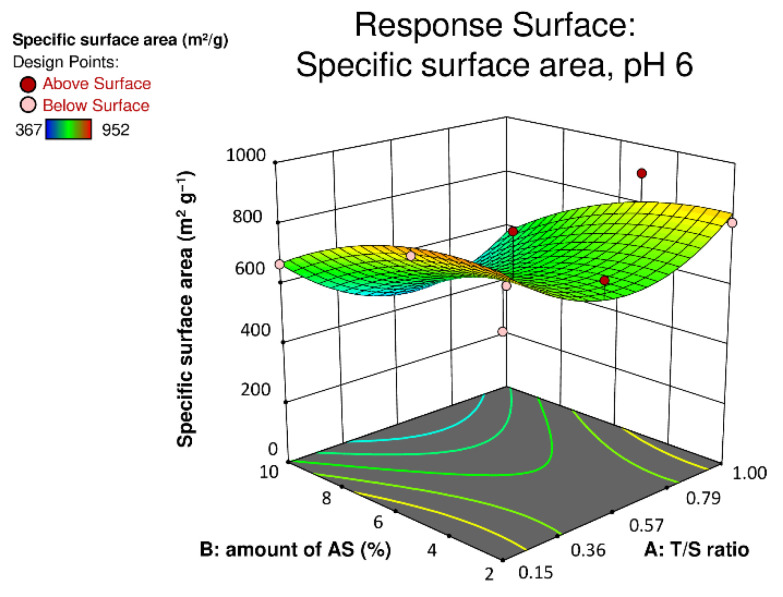
Specific surface area of the tannin-silica hybrid aerogels as a function of their composition at a pH value of 6.

In order to analyze the nitrogen isotherms regarding their influence of the amount of crosslinker on the specific surface area as well as the mesopore volume in greater detail, statistical analysis has been carried out. Further information regarding the statistical evaluation can be found in the supporting information. Analysis of variance (ANOVA) was carried out to be able to determine significant factors of this model (Supporting Information, Figure S7). It was found that the amount of used crosslinker is significant. This implies that solely the amount of crosslinker influences the resultant materials’ specific surface area strongly. In contrast to that the T/S ratio as well as the pH values used have no significant influence on the specific surface. The statistical analysis allows visualization of the effect of the investigated factors on the specific surface area of the hybrid material, within the scope of a so-called response surface. The response surface, generated at a pH value of 6 and using the factor variables of the T/S ratio as well as the amount of crosslinker used in correspondence to the specific surface area of the resultant material is depicted in Figure 8. In order to stress the difference between low and high specific surface areas of the TS hybrid materials, a color code, from blue to red has been used. Moreover, the red dots portray the actual measured specific surface areas for the precise formulations. It can clearly be seen that a high amount of crosslinker (i.e., 10 wt%) yields the lowest values for the specific surface area of the material, independent on the T/S ratio used. Furthermore, the highest surface areas gained are observed for a medium amount of crosslinker (i.e., 5 wt%). Thus, in order to gain a highly porous tannin-silica hybrid aerogel with a high specific surface area, which is desired for various applications, the optimal amount of used crosslinker is 5 wt%. In addition, it seems that a T/S ratio of 0.5 features comparably lower specific surface areas of the resultant materials; however, according to ANOVA, the T/S ratio depicts no significant factor. Nevertheless, these results only account in correspondence to the specific surface area since for example the T/S ratio indeed influences the amount of retained tannin, as determined earlier.

Furthermore, statistical analysis using the same model was carried out to evaluate the obtained results regarding the materials’ mesopore volume. The mesopore volume has been determined up to 40 nm and, hence, macropores were not included in the analysis. First, to determine significant factors of the model, ANOVA has been carried out (Supporting Information, Figure S8). It was found that the amount of crosslinker used, the pH value as well as the variance of the T/S ratio have a significant influence on the mesopore volume. The response surface, generated at a pH value of 6 and using the factor variables of the T/S ratio as well as the amount of crosslinker used in correspondence to the mesopore volume of the resultant material is depicted in Figure S9.

In regard to the amount of crosslinker used, the mesopore volume shows, as clearly can be seen in the response surface, similar behavior as the one for the specific surface area. More precise, an amount of 5 wt% crosslinker used, exhibits materials with the highest mesopore volumes. Furthermore, the red areas of the response surface stress, that a lower T/S ratio of 0.15 yields materials with higher mesopore volume. Hence, also the T/S ratio shows a certain dependency on the materials’ mesopore volume. Moreover, it has to be mentioned that the pH value, as already determined using ANOVA, has a significant influence on the mesopore volume, since in general the gels prepared at a lower pH feature distinctly lower mesopore volumes.

Overall, several parameters influence different characteristics of the generated tannin-silica network. More precise, by solely varying the pH value, the gelation behavior, i.e., gelation time, of the tannin-silica batches can be influenced. Additionally, the pH value influences the mesopore volume of the resultant hybrid material. Furthermore, the T/S ratio has as well a significant effect on the resultant hybrid materials’ mesopore volume. In addition, in particular, the amount of crosslinker used has to be stressed, as it has a significant influence on the amount of retained tannin as well as on the materials’ properties, i.e., specific surface area and mesopore volume. However, 5 wt% of crosslinker used, generally evokes materials with improved properties. In addition, the more crosslinker is used, the more tannin has been retained in the hybrid network. In conclusion, this study provides first insights on the one-pot synthesis of tannin-silica hybrids regarding the processing parameter design.

### 3.3. Thermal Treatment of Hybrid Tannin-Silica Networks

**Figure 9:**
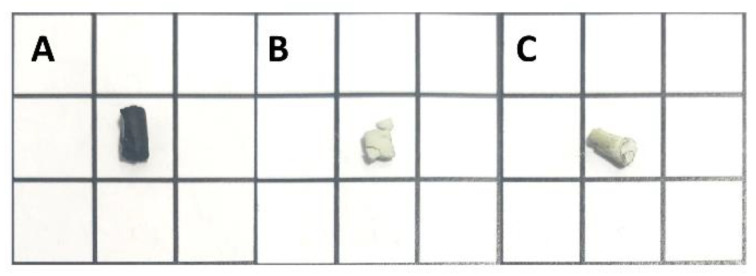
Photographs of the carbonized hybrid gels TS_0_._15_6_10_c_ (**A**), TS_0_._5_6_10_c_ (**B**), TS_1_._0_6_10_c_ (**C**), illustrated on a 1 cm^2^ grid.

Silicon carbide materials are widely recognized in various applications, e.g., as absorbent or catalysts, due to the materials’ properties including high thermal conductivity and high mechanical stability [32]. Hence, a desirable characteristic of the tannin-silica hybrid material should be its suitability to act as bio-based precursor for silicon carbide. In order to achieve a complete conversion of SiO_2_ to SiC a C:SiO_2_ stoichiometric ratio of 1:3 is required, as proposed in literature [46]. The obtained tannin-silica hybrid aerogels were carbonized and analyzed regarding their chemical structure using X-ray diffraction (XRD) measurements as well as Raman spectroscopy. Specifically, monoliths of the batches TS_0_._15_6_10_, TS_0_._5_6_10_ and TS_1_._0_6_10_ were carbonized. These batches have been selected as they solely differ in their T/S ratio, whereby they all feature the same processing pH value and the same amount of crosslinker. While taking solely the amount of retained tannin in the network into account, the tannin-silica aerogels TS_0_._15_6_10_, TS_0_._5_6_10_ and TS_1_._0_6_10_ feature a T/SiO_2_ ratio of 0.2, 1 and 2, respectively. Thus, the impact of the T/SiO_2_ ratio on the generation of silicon carbide materials are hereinafter discussed and photographs of the carbonized monoliths are displayed in Figure 9.

A remarkably great difference in the color of the carbonized gels can be observed, whereas the gel of the batch of TS_0_._15_6_10_c_ appears black and, in contrast to that, the other two carbonized gels with a higher T/S ratio appear whitish. This difference in color might already be the first sign for different phase compositions of the carbonized gels. 

**Figure 10:**
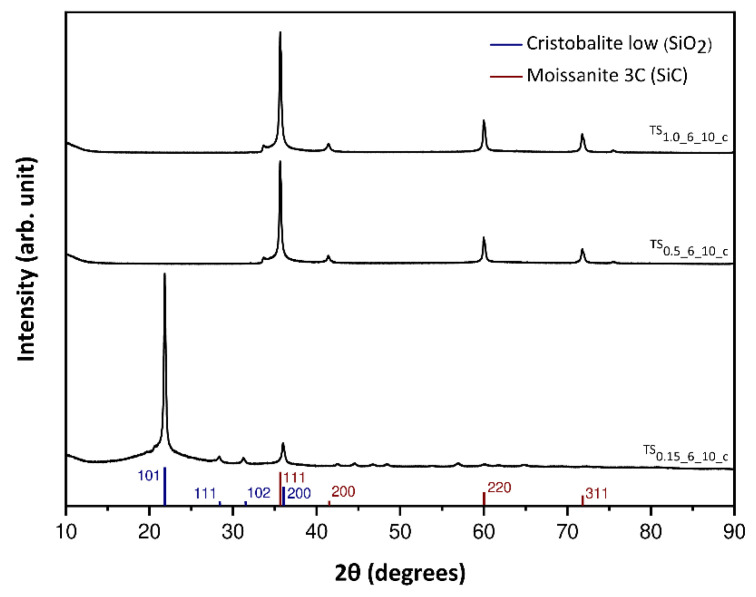
XRD patterns of the samples TS_0_._15_6_10_c_, TS_0_._5_6_10_c_ and TS_1_._0_6_10_c_ after carbothermal treatment under argon atmosphere at 1500 °C for 5 h.

The XRD patterns of the carbonized samples (Figure 10) are similar for the samples TS_0_._5_6_10_c_ and TS_1_._0_6_10_c_, whereas the one of TS_0_._15_6_10_c_ differs distinctly. Based on a database search using the software EVA V5.0, the batches TS_0_._5_6_10_c_ and TS_1_._0_6_10_c_ pertain with high certainty silicon carbide (moissanite 3C; ISCD Collection Code: 28389) [47]. Thereby, the identification is made because of the agreement of the major reflection plane at roughly 2ϴ = 35.7° (111, d = 2.5 Å), as well as minor reflection planes at approximately 2ϴ = 41.4° (200, d = 2.2 Å), 2ϴ = 60.0° (220, d = 1.5 Å) and 2ϴ = 71.8° (311, d = 1.3 Å). In comparison to that, the XRD pattern of TS_0_._15_6_10_c_ can be assigned to silicon dioxide (cristobalite low; ISCD Collection Code: 9327) with a large reflection plane at 2ϴ = 21.8° (101, d = 4.1 Å) [48]. Furthermore, between the 2ϴ values of approximately 16 to 26° an elevated broadening of the spectrums’ baseline can be observed, indicating the presence of an amorphous phase, presumably amorphous SiO_2_. Furthermore, minor reflection planes were found that match the silicon dioxide at 2ϴ = 28.4° (111, d = 3.1 Å), 2ϴ = 31.4° (102, d = 2.9 Å) and 2ϴ = 36.0° (200, d = 2.5 Å). Whereby, the latter reflection plane could feature superimpositions with the silicon carbides’ major reflection plane at 2ϴ = 35.7° (111, d = 2.5 Å). In order to analyze the phase composition of the carbonized material of the batch TS_0_._15_6_10_c_, quantification employing the moissanite 3C [47] as well as the cristobalite [48] structure as reference, was carried out using the TOPAS software. The results of the quantification analysis show that the carbonized material of the batch TS_0_._15_6_10_c_ is a composite material made up of approximately 5% moissanite as well as 95% cristobalite (Supporting Information, Figure S10). Therefore, the generation of a silicon carbide material is only accomplished rudimentarily for carbonized tannin-silica hybrid aerogels with a T/S ratio of 0.15 (T/SiO_2_ ratio of 0.2). More precise, the lower the T/S ratio, the lower the amount of organic material in the composite. Hence, composites with more organic precursor (e.g., T/S ratios of 0.5 and 1.0; T/SiO_2_ ratios of 1 and 2, respectively) yield a pure silicon carbide material after carbothermal reduction. Thus, it can be concluded that a T/S ratio of 0.15 features a too low stoichiometric ratio of C:SiO_2_, hence, solely allowing the generation of a (composite) material, consisting of minor traces of SiC and majoritarian SiO_2_. In order to verify this, Raman spectra were recorded (Supporting Information, Figure S11). In agreement with the XRD results, Raman spectroscopic measurements suggest a silicon carbide (3C) material for the samples TS_0_._5_6_10_c_ and TS_1_._0_6_10_c_, indicated by the distinct band at roughly 790 cm^−1^ [31]. The spectrum of TS_0_._15_6_10_c_ (Figure S11A) depicts the structure of cristobalite with representative bands at approximately 415, 230 and 110 cm^−1^ [49,50]. Furthermore, the minor band at roughly 785 cm^−1^ either arises from the cristobalite structure or rather from possible SiC structure or presents its superimposition. However, the Raman spectroscopic measurement most likely agrees with XRD results that only minor traces of SiC are present. Moreover, the distinct D- and G-band, at a wavenumber of 1338 and 1600 cm^−1^, respectively, indicate the presence of carbon. Thus, this composite consists mainly of carbon, whose structure cannot be resolved within XRD as its crystalline size (roughly 3 nm, suggested by TEM) succumbs the XRD resolution limit, cristobalite and minor traces of SiC. More precise, this crystalline carbon structure could be indicated within the X-ray amorphous region (16° to 26°) within the X-ray pattern of the TS_0_._15_6_10_c_ aerogel (Figure 10).

Overall, this study supports the applicability of the bio-based tannin-silica aerogel system with a T/S ratio of 0.5 and 1.0 to function as precursor for silicon carbide composite materials and, hence, be able to replace currently utilized SiC materials, nowadays commonly made up of toxic RF and non-water-soluble alkoxysilanes, in various applications. Nevertheless, further research needs to be done in order to increase the yield of silicon carbide as well as to improve its resultant monolithic structure.

## 4. Conclusions

This study presents a novel one-pot approach in aqueous media, allowing the generation of porous tannin-silica hybrid aerogels, finding application as bio-based precursor alternative to the commercially available environmentally harmful silicon carbide precursor materials. Yielding a covalent tannin-silica network, requires the use of an aldehyde-functionalized silane as crosslinker, i.e., triethoxysilylbutyraldehyde, which benefits the retention of tannin. The synthesized tannin-silica hybrids, hence, feature a silica macrostructure with homogeneously incorporated tannin as verified by solid state NMR and STEM imaging.

Generating the hybrid material allows the combination of the favorable properties of the organic and inorganic precursors, namely mechanical stability and high specific surface areas, respectively. Hence, within this systematic study, several process parameters, namely the pH value, the T/S ratio as well as the amount of used crosslinker, have been altered. The obtained results regarding the materials’ properties have been statistically analyzed using ANOVA as well as response surfaces, which illustrate the influences of the above-named parameters on the resulting characteristics of the monoliths, i.e., pore volume and specific surface area. It was found that in particular the amount of crosslinker has a significant influence on the materials’ properties. More precisely, the usage of 5 wt% of crossslinker results in tannin-silica materials with high specific surfaces of roughly 825–900 m^2^ g^−1^, as well as with high mesopore volumes (1.4–2.0 g cm^−3^) for a pH value of 6. 

Overall, this study provides important first information about the generation of a sustainable hybrid system made up of tannin and silica and their suitability for bio-based applications.

## Figures and Tables

**Table 1:** Determined shrinkage percentage, porosity, bulk density, skeletal density values, mesopore volume (* up to 40 nm) and specific surface area of the prepared tannin-silica aerogels using a crosslinker.

Batch	Shrinkage (%)	Porosity (%)	Bulk Density (g cm^−3^)	Skeletal Density (g cm^−3^)	Mesopore Volume * (g cm^−3^)	S_BET_ (m^2^ g^−1^)
TS_0_._15_3_2_	7	93	0.12	1.58	1.9	952
TS_0_._15_3_5_	10	90	0.16	1.49	1.0	828
TS_0_._15_3_10_	6	88	0.18	1.43	0.2	416
TS_0_._5_3_2_	12	92	0.12	1.48	0.8	722
TS_0_._5_3_5_	11	91	0.13	1.43	0.3	367
TS_1_._0_3_2_	10	94	0.09	1.51	0.6	557
TS_0_._15_6_2_	1	95	0.10	1.80	1.5	692
TS_0_._15_6_5_	2	94	0.10	1.71	2.0	830
TS_0_._15_6_10_	2	94	0.09	1.61	1.6	673
TS_0_._5_6_2_	5	96	0.07	1.67	1.4	750
TS_0_._5_6_5_	6	95	0.07	1.47	1.4	823
TS_0_._5_6_10_	5	94	0.10	1.68	0.9	600
TS_1_._0_6_2_	6	95	0.07	1.45	0.9	811
TS_1_._0_6_5_	5	94	0.09	1.48	1.7	905
TS_1_._0_6_10_	5	94	0.09	1.49	0.5	385

## Supplementary Materials

The following are available online at https://www.mdpi.com/article/10.3390/ma14185231/s1, Figure S1: Nomenclature of synthesized tannin-silica aerogels, Table S1: Amounts employed for generation of the tannin-silica hybrid aerogels, Figure S2: Photographs, illustrated on a 1 cm^2^ grid, of the tannin-silica hybrid gels TS_0_._15_3_ (A), TS_0_._5_3_ (B), TS_1_._0_3_ (C), TS_0_._15_6_ (D), TS_0_._5_6_ (E) and TS_1_._0_6_ (F) after supercritical drying, Table S2: Determined gelation times, shrinkages as well as amount of retained tannin of the tannin-silica aerogels, synthesized without the usage of a crosslinker, Figure S3: Representative TGA curve of an aerogel of the batch TS_0_._15_3_, Figure S4: Schematic sketch of the presumed structure of the crosslinked tannin-silica aerogels using an aldehyde-functionalized silane as crosslinker, Table S3: Gelation time ranges of the synthesized TS batches, using AS as a crosslinker, according to their pH value as well as their T/S ratio, Figure S5: Structure and peak assignment of mimosa tannin (left) and triethoxysilylbutyraldehyde (right), Table S4: ^1^H-^13^C peak assignments, Figure S6: ^1^H-^29^Si CP MAS NMR spectra, normalized to the same maximum peak intensity and their deconvolution: black dots (experimental data), black line (fitting envelope), blue lines (fitted Gaussian components), red lines (fit residual, Table S5: Retained amounts of tannin for the tannin-silica hybrid aerogel system, Figure S7: ANOVA spreadsheet, whereat the specific surface area is set as response, Figure S8: ANOVA spreadsheet, whereat the mesopore volume is set as response, Figure S9 Mesopore volume of the tannin-silica hybrid aerogels as a function of their composition at a pH value of 6, Figure S10 Quantification of phase composition of TS_0_._15_6_10_c_ using Moissanite 3C (SiC) and Cristobalite (SiO_2_) as reference and by utilizing the TOPAS software, Figure S11 Raman spectra of the carbonized TS_0_._15_6_10_c_ (black), TS_0_._5_6_10_c_ (red) and TS_1_._0_6_10_c_ (green), recorded by utilizing a 532 nm laser wavelength and a power of 4 mW.
