# Supplementary material for: A Systematic Study on Bio-Based Hybrid Aerogels Made of Tannin and Silica
Source: Materials (Basel). 2021 Sep 11;14(18):5231. doi: 10.3390/ma14185231 (PMC8468457; doi:10.3390/ma14185231)
Supplement: Supplementary file 1 [file materials-14-05231-s001.zip › materials-1354832-supplementary.pdf]

Supporting Information

A Systematic Study on Bio-Based Hybrid Aerogels Made of Tannin and Silica

Ann-Kathrin Koopmann[1,2], Wim J. Malfait[3], Thomas Sepperer[2,4] and Nicola Huesing[1,2,*]

[1] Paris-Lodron University of Salzburg, Department of Chemistry and Physics of Materials, 5020 Salzburg, Austria.

[2] Salzburg Center for Smart Materials, 5020 Salzburg, Austria.

[3] Swiss Federal Laboratories for Materials Science and Technology, EMPA 8600 Dübendorf, Switzerland.

[4] Salzburg University of Applied Sciences, Forest Products Technology and Timber Constructions, 5431 Kuchl, Austria.

[*] Corresponding author: nicola.huesing@sbg.ac.at

Additional information on the synthesis of tannin-silica hybrid aerogels:

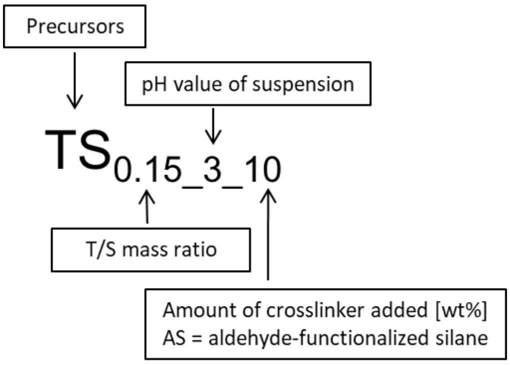

Figure S1 Nomenclature of synthesized tannin-silica aerogels.

Table S1 Amounts employed for generation of the tannin-silica hybrid aerogels.

Batch	Tannin (g)	EGMS (g)	H_2O (g)	Crosslinker AS (µL)	pH value
**$TS_{0.15\_3}$**	0.25	1.68	6.20	/	3
**$TS_{0.5\_3}$**	0.42	0.84	6.00	/	3
**$TS_{1.0\_3}$**	0.42	0.42	5.10	/	3
**$TS_{0.15\_6}$**	0.25	1.68	6.20	/	6
**$TS_{0.5\_6}$**	0.42	0.84	6.00	/	6
**$TS_{1.0\_6}$**	0.42	0.42	5.10	/	6
**$TS_{0.15\_3\_2}$**	0.25	1.68	6.20	170	3
**$TS_{0.15\_3\_5}$**	0.25	1.68	6.20	423	3
**$TS_{0.15\_3\_10}$**	0.25	1.68	6.20	847	3
**$TS_{0.5\_3\_2}$**	0.42	0.84	6.00	152	3
**$TS_{0.5\_3\_5}$**	0.42	0.84	6.00	380	3
**$TS_{0.5\_3\_10}$**	0.42	0.84	6.00	760	3
**$TS_{1.0\_3\_2}$**	0.42	0.42	5.10	124	3
**$TS_{1.0\_3\_5}$**	0.42	0.42	5.10	310	3
**$TS_{1.0\_3\_10}$**	0.42	0.42	5.10	621	3
**$TS_{0.15\_6\_2}$**	0.25	1.68	6.20	170	6
**$TS_{0.15\_6\_5}$**	0.25	1.68	6.20	423	6
**$TS_{0.15\_6\_10}$**	0.25	1.68	6.20	847	6
**$TS_{0.5\_6\_2}$**	0.42	0.84	6.00	152	6
**$TS_{0.5\_6\_5}$**	0.42	0.84	6.00	380	6
**$TS_{0.5\_6\_10}$**	0.42	0.84	6.00	760	6
**$TS_{1.0\_6\_2}$**	0.42	0.42	5.10	124	6
**$TS_{1.0\_6\_5}$**	0.42	0.42	5.10	310	6
**$TS_{1.0\_6\_10}$**	0.42	0.42	5.10	621	6

Tannin-silica network without the usage of a crosslinker:

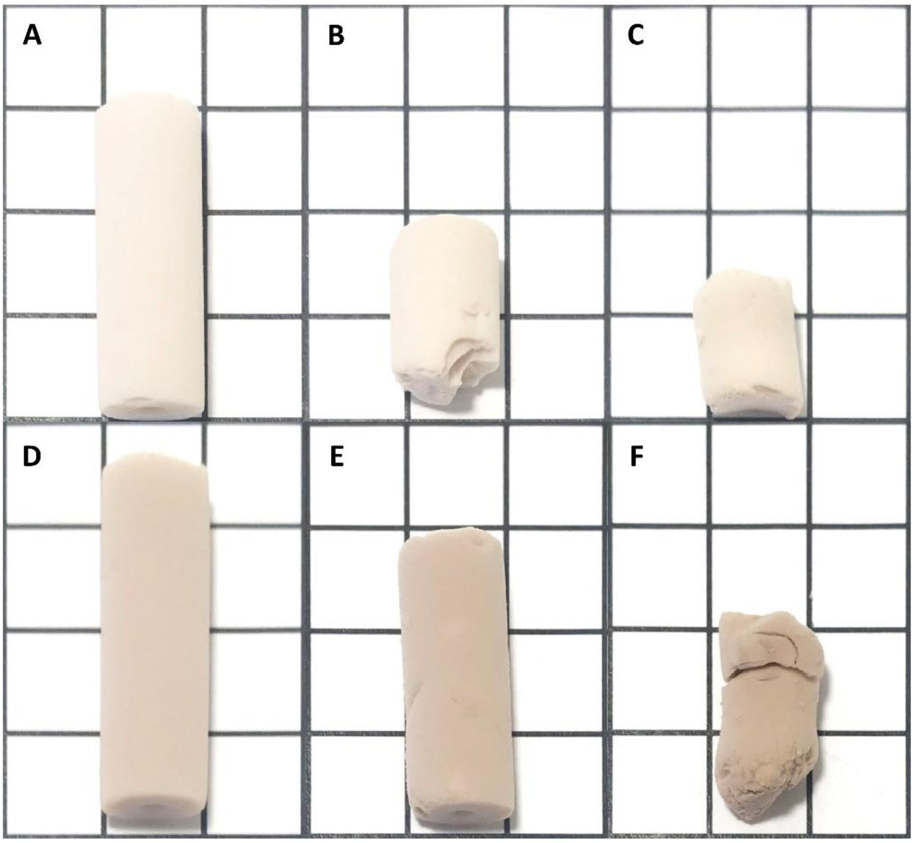

Figure S2 Photographs, illustrated on a 1 cm² grid, of the tannin-silica hybrid gels $TS_{0.15\_3}$ (A), $TS_{0.5\_3}$ (B), $TS_{1.0\_3}$ (C), $TS_{0.15\_6}$ (D), $TS_{0.5\_6}$ (E) and $TS_{1.0\_6}$ (F) after supercritical drying**.**

Table S2 Determined gelation times, shrinkages as well as amount of retained tannin of the tannin-silica aerogels, synthesized without the usage of a crosslinker.

Batch	Gel time	Shrinkage (%)	Amount of retained tannin (%)
$\mathbf{TS_{0.15\_3}}$	1 h 12min	2.4	11
$\mathbf{TS_{0.5\_3}}$	1 h 15 min	5.0	5
$\mathbf{TS_{1.0\_3}}$	~ 18 h	12.6	4
$\mathbf{TS_{0.15\_6}}$	12 min	2.4	11
$\mathbf{TS_{0.5\_6}}$	20 min	3.2	7
$\mathbf{TS_{1.0\_6}}$	23 min	12.7	5

Additional information concerning the TG-analysis of tannin-silica gels

The synthesized aerogels were analyzed for their amount of extracted tannin using thermogravimetric analysis (TGA). The TGA curves of all synthesized tannin-silica hybrid aerogels show similar curve behavior, nevertheless they differ in their residual mass of SiO_2. Hence, a representative TGA curve is illustrated in Figure S3.

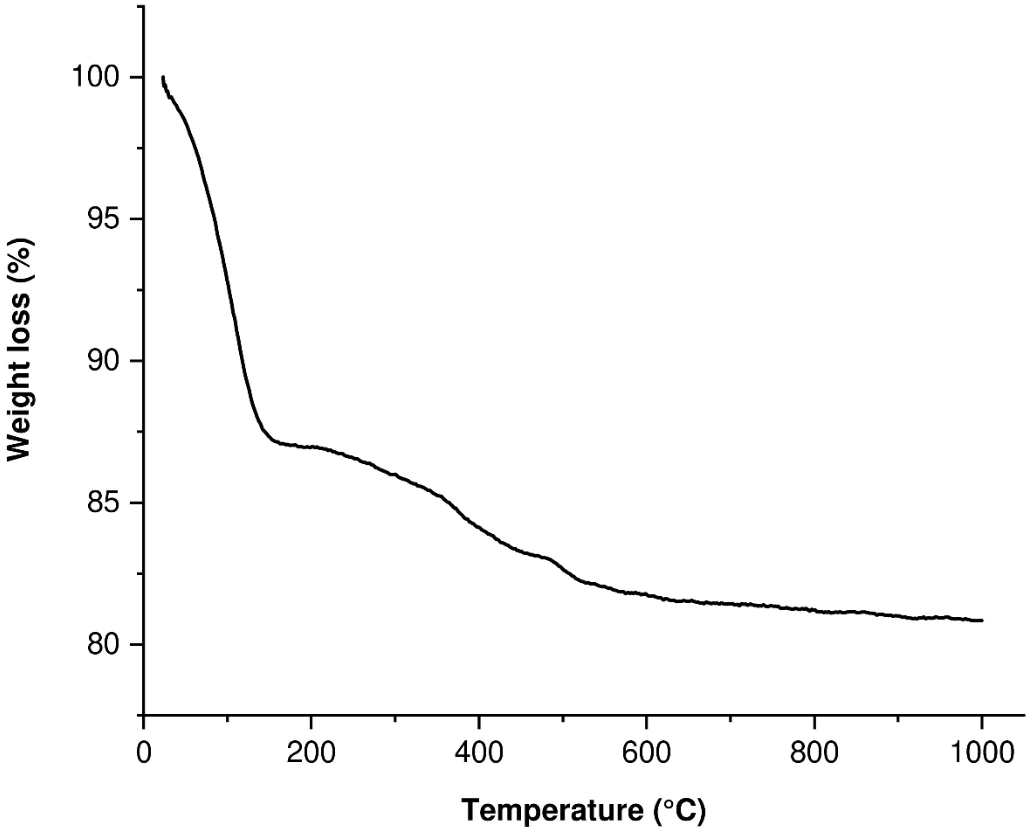

Figure S3 Representative TGA curve of an aerogel of the batch $TS_{0.15\_3}$.

The TGA curve of the batch $TS_{0.15\_3}$ shows weight loss of roughly 15% between room temperature and 100 °C accounts for the loss of water, as the tannin is a hygroscopic reagent, which absorbs water from the atmosphere even after the supercritical drying process. Thus, the retained aerogels have either be stored in a desiccator in order to prevent this weight loss in the TGA curve or alternatively, the TGA results can be normalized by subtracting the weight loss due to absorbed water to allow accurate calculation of the amount of extracted tannin based on the normalized residual amount of SiO_2. Furthermore, a mass decrease can be observed at a temperature between roughly 200 and 800 °C, which indicates the decomposition of the organic residues, which reside on the surface of the aerogel particles' functional groups. Furthermore, it has to be noted that all synthesized TS aerogels have been washed during the solvent exchange step with ethanol. Thus, it is possible that ethoxy-groups reside on the surface of the nanoparticles, which generate the gel-network. Hence, the decomposition of the ethoxy-groups can be seen in the TGA curve as well starting at a temperature of 130 °C.

Calculation of retained tannin using TGA analysis

The amount of retained tannin during the process of aerogel generation is determined under the assumption EGMS and the crosslinker are entirely incorporated into the resulting network and not lost during solvent exchange or supercritical drying. The investigation of the tannin-silica gel network using TGA gives the residual amount of SiO_2 within the gel network, which stems from EGMS and the aldehyde-functionalized silane crosslinker, whose residual masses are known due to prior TGA of the pure components. Calculating a theoretical residual mass fraction ($m_{res,th}$) of the tannin-silica gel is possible using eq. (1), where m_{EGMS}, m_{cl},$m_{res,EGMS}$, $m_{res,\mathrm{cl}}$ and m_{tan} are the incorporated mass of EGMS and crosslinker as well as the residual mass of EGMS, the crosslinker and of tannin, respectively.

$$m_{res,th} = \frac{m_{EGMS} + m_{cl}}{m_{res,EGMS} + m_{res,cl} + m_{tan}} \cdot 100\ [\%]$$	Eq. (1)

Comparing the theoretical residual mass with the actual residual mass ($m_{res,TGA}$) from TGA, the amount of extracted tannin can be calculated using eq. (2) where $m_{res,tan}$ is the remaining amount of tannin in the gel calculated by eq. (3) where k presents the percentage of extracted tannin acquired using the target factor analysis by excels solver function. The percentage of retained tannin is calculated by 100% minus the percentage of extracted tannin.

$$m_{res,TGA} = \frac{m_{EGMS} + m_{cl}}{m_{res,EGMS} + m_{res,cl} + m_{res,tan}} \cdot 100\ [\%]$$	Eq. (2)

$$m_{res,tan} = m_{tan} \cdot (1 - k)$$	Eq. (3)

Note: This calculation solely presents an approximation of the amount of retained tannin, as the minor amounts of organic residues of the crosslinker, present in the tannin-silica network, have been neglected within this calculation. Nevertheless, tendency of the results is valid and amount of retained tannin is only negligible lower.

Tannin-silica network using a crosslinker:

Proposed bonding situation of the tannin-silica hybrid aerogels

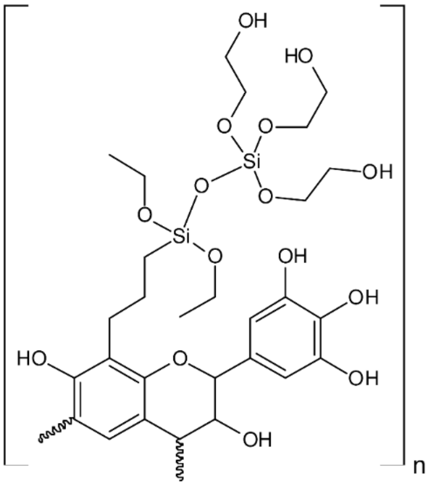

Figure S4 Schematic sketch of the presumed structure of the crosslinked tannin-silica aerogels using an aldehyde-functionalized silane as crosslinker.

Table S3 Gelation time ranges of the synthesized TS batches, using AS as a crosslinker, according to their pH value as well as their T/S ratio.

pH value	T/S ratio	Gel time
3	0.15	2.3-3.8 h
3	0.50	3.6-4.3 h
3	1.0	22 h
6	0.15	15-19 min
6	0.50	17-28 min
6	1.0	21-30 min

Additional information concerning NMR measurements

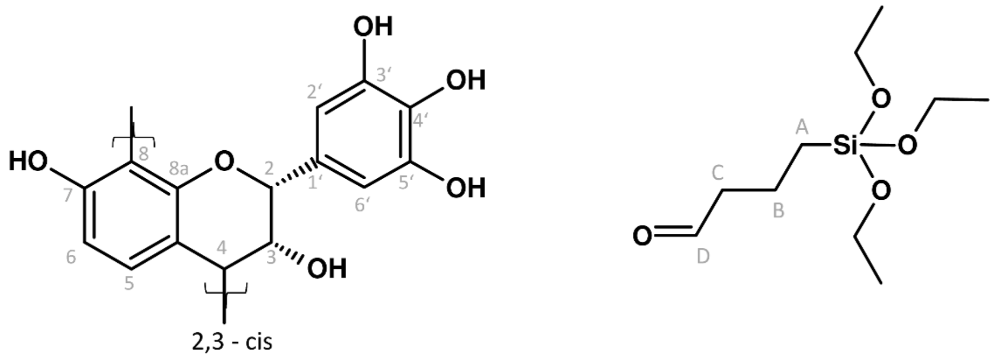

Figure S5 Structure and peak assignment of mimosa tannin (left) and triethoxysilylbutyraldehyde (right).

Table S4 ^{1}H-^{13}C peak assignments.

Peak	v(F1) (ppm)	Molecule	Assignment
**1**	207	-silyl-butyraldehyde	D
**2**	194.7	tannin	ssb to peak 5
**3**	185.2	tannin	ssb to peak 6
**4**	170.9	tannin	ssb to peak 7
**5**	154.2	tannin	C5, C7, C8a
**6**	145.3	tannin	C3', C4'
**7**	132.2	tannin	C1
**8**	114.9	tannin	ssb to peak 5
**9**	108.4	tannin	C4a, C6, C8, C2', C5', C6'
**10**	104.7		
**11**	91.2	tannin	ssb to peak 7
**12**	83-60 (broad)	tannin	C2, C3
**13**	64.6		
**14**	57.9	ethoxy	CH_2
**15**	46.2	unreacted -silyl-butyraldehyde	C
**16**	41.5	tannin (linked)	C4
**17**	36.1	reacted -silyl-butyraldehyde	C
**18**	32.4	tannin (non-linked)	C4
**19**	~16 (shoulder)	ethoxy	CH_3
**20**	15.2	-silyl-butyraldehyde	B
**21**	~13 (shoulder)	-silyl-butyraldehyde	A

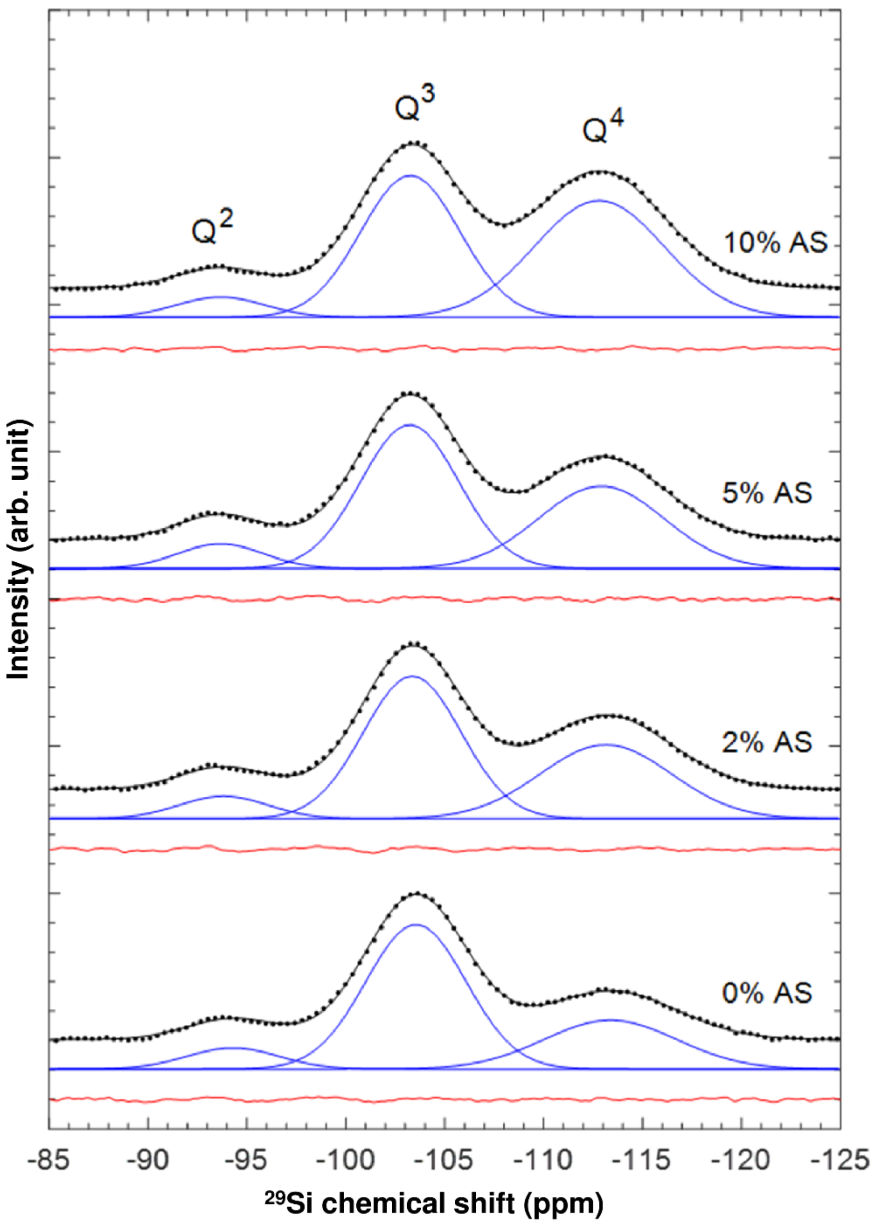

Figure S6 ^{1}H-^{29}Si CP MAS NMR spectra, normalized to the same maximum peak intensity, and their deconvolution: black dots (experimental data), black line (fitting envelope), blue lines (fitted Gaussian components), red lines (fit residual). The spectra are offset vertically for clarity.

Table S5 Retained amounts of tannin for the tannin-silica hybrid aerogel system.

Batch	Retained amount of tannin (%)	Initial amount of tannin (g)	Amount of tannin present in the gel network (g)
**$TS_{0.15\_3}$**	11	0.25	0.03
**$TS_{0.5\_3}$**	5	0.42	0.02
**$TS_{1.0\_3}$**	4	0.42	0.02
**$TS_{0.15\_6}$**	11	0.25	0.03
**$TS_{0.5\_6}$**	7	0.42	0.03
**$TS_{1.0\_6}$**	5	0.42	0.02
**$TS_{0.15\_3\_2}$**	51	0.25	0.13
**$TS_{0.15\_3\_5}$**	89	0.25	0.22
**$TS_{0.15\_3\_10}$**	97	0.25	0.24
**$TS_{0.5\_3\_2}$**	40	0.42	0.17
**$TS_{0.5\_3\_5}$**	47	0.42	0.20
**$TS_{1.0\_3\_2}$**	30	0.42	0.13
**$TS_{0.15\_6\_2}$**	27	0.25	0.07
**$TS_{0.15\_6\_5}$**	46	0.25	0.12
**$TS_{0.15\_6\_10}$**	89	0.25	0.22
**$TS_{0.5\_6\_2}$**	24	0.42	0.10
**$TS_{0.5\_6\_5}$**	37	0.42	0.16
**$TS_{0.5\_6\_10}$**	43	0.42	0.18
**$TS_{1.0\_6\_2}$**	21	0.42	0.09
**$TS_{1.0\_6\_5}$**	32	0.42	0.13
**$TS_{1.0\_6\_10}$**	38	0.42	0.16

Statistical evaluation of the material characteristics of the tannin-silica aerogels crosslinked with an aldehyde-functionalized silane

Overall, the software Design Expert has been used to statistically analyze the material properties of the tannin-silica aerogels. The generation of the statistical model for the result interpretation of the tannin-silica aerogel system has been carried out by defining the T/S ratio, the amount of crosslinker and the pH value as numeric factors as well as the specific surfaces area as response. Furthermore, the main factors and also the two-way factor interactions have been analyzed. The statistical model was designed by using a quadratic process order and a polynominal model type. By applying the quadratic process order to the model it was found that the model is aliased, in particular the variance of the pH value has been aliased. Thus, in order to carry out a non-aliased model the factor variance of the pH value (C^2) has been left out for further analysis.

Specific surface area

ANOVA for Reduced Quadratic model

Response 1: specific surface area

Source	Sum of Squares	df	Mean Square	F-value	p-value	
**Model**	4,215E+05	8	52689,69	2,30	0,1081	not significant
A-T/S ratio	467,99	1	467,99	0,0205	0,8891	
B-amount of AS	2,627E+05	1	2,627E+05	11,49	0,0069	
C-pH value	8042,58	1	8042,58	0,3517	0,5663	
AB	1885,54	1	1885,54	0,0825	0,7799	
AC	8821,26	1	8821,26	0,3857	0,5484	
BC	33794,22	1	33794,22	1,48	0,2520	
A^2	71427,50	1	71427,50	3,12	0,1076	
B^2	14627,77	1	14627,77	0,6397	0,4424	
**Residual**	2,287E+05	10	22867,84			
Lack of Fit	2,287E+05	6	38113,07			
Pure Error	0,0000	4	0,0000			
**Cor Total**	6,502E+05	18				

Factor coding is **Coded**.
Sum of squares is **Type III - Partial**

The **Model F-value** of 2,30 implies the model is not significant relative to the noise. There is a 10,81% chance that an F-value this large could occur due to noise.

P-values less than 0,0500 indicate model terms are significant. In this case B is a significant model term. Values greater than 0.1000 indicate the model terms are not significant. If there are many insignificant model terms (not counting those required to support hierarchy), model reduction may improve your model.

Figure S7 ANOVA spreadsheet, whereat the specific surface area is set as response.

Note: Response surface generation

Solely the response surface of a processing pH value of 6 is presented, since not all batch formulation with a pH value of 3 gelled and hence the specific surface area would only allow improper interpretation.

Mesopore volume

ANOVA for Reduced Quadratic model

Response 1: total pore volume

Source	Sum of Squares	df	Mean Square	F-value	p-value	
**Model**	4,94	8	0,6169	4,20	0,0189	significant
A-T/S ratio	0,2241	1	0,2241	1,53	0,2450	
B-amount of AS	1,25	1	1,25	8,50	0,0154	
C-pH value	0,8443	1	0,8443	5,75	0,0375	
AB	0,0426	1	0,0426	0,2899	0,6021	
AC	0,2703	1	0,2703	1,84	0,2048	
BC	0,4377	1	0,4377	2,98	0,1150	
A^2	0,8378	1	0,8378	5,70	0,0381	
B^2	0,1376	1	0,1376	0,9369	0,3559	
**Residual**	1,47	10	0,1469			
Lack of Fit	1,47	6	0,2448			
Pure Error	0,0000	4	0,0000			
**Cor Total**	6,40	18				

Factor coding is **Coded**.
Sum of squares is **Type III - Partial**

The **Model F-value** of 4,20 implies the model is significant. There is only a 1,89% chance that an F-value this large could occur due to noise.

P-values less than 0,0500 indicate model terms are significant. In this case B, C, A^2 are significant model terms. Values greater than 0.1000 indicate the model terms are not significant. If there are many insignificant model terms (not counting those required to support hierarchy), model reduction may improve your model.

Figure S8 ANOVA spreadsheet, whereat the mesopore volume is set as response.

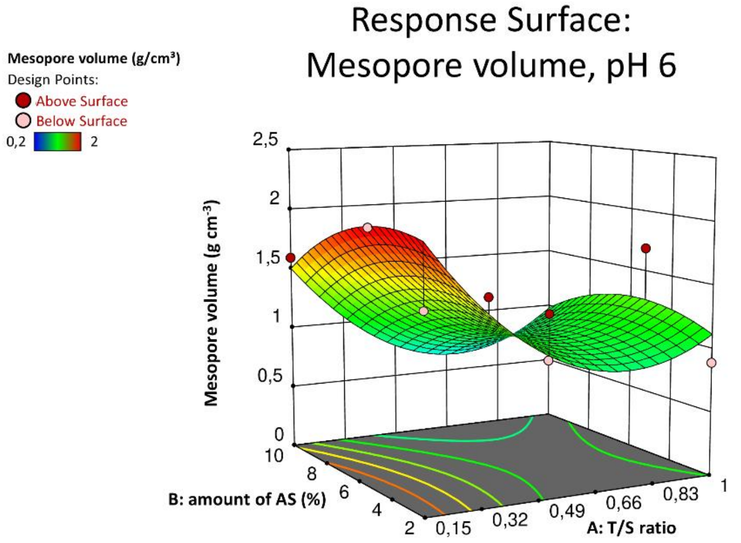

Figure S9 Mesopore volume of the tannin-silica hybrid aerogels as a function of their composition at a pH value of 6.

Quantification of the gained XRD results after carbothermal reduction of the tannin-silica aerogel

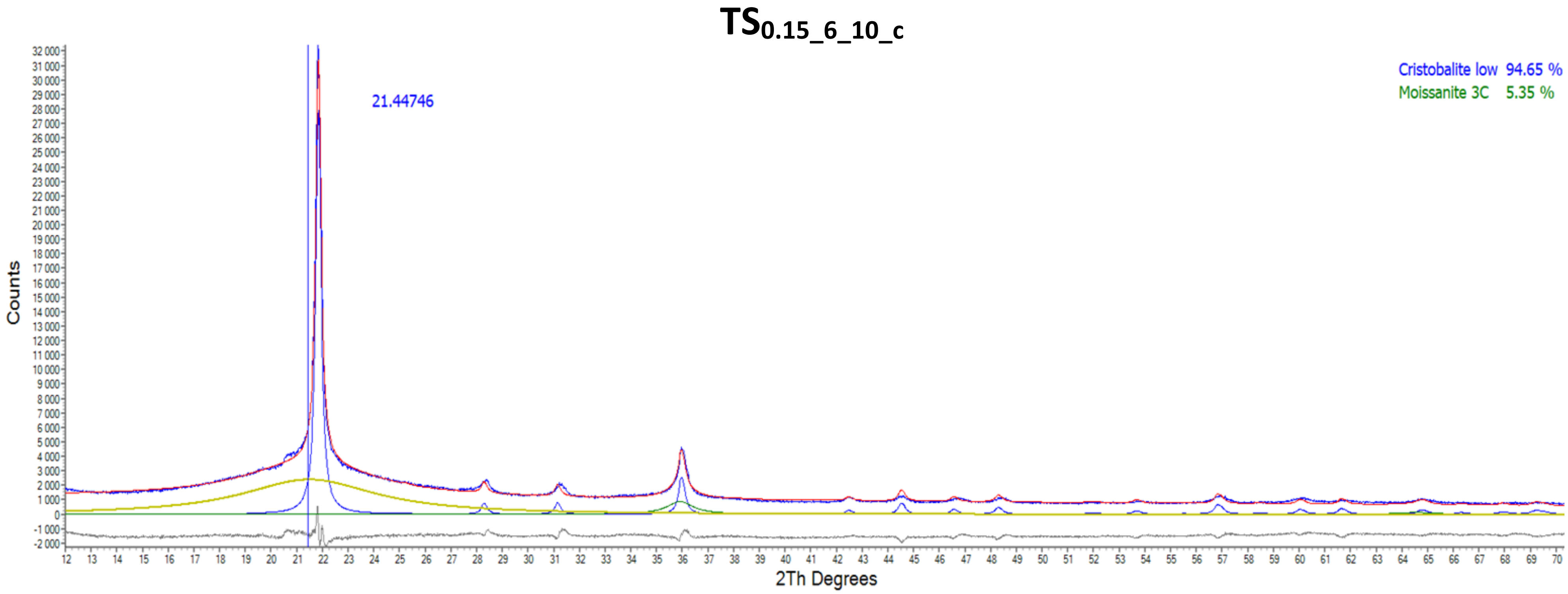

Figure S10 Quantification of phase composition of $TS_{0.15\_6\_10\_c}$ using Moissanite 3C (SiC) and Cristobalite (SiO_2) as reference and by utilizing the TOPAS software. The blue graph depicts the samples raw data, whereby the red graph shows the fitted data. The thin blue curve represents the XRD pattern of Cristobalite, the thin green curve the XRD pattern of Moissanite and the yellow curve the peak phase, which is included at 21.4° to fit the amorphous part of the XRD pattern.

Additional information concerning the Raman measurements after carbothermal reduction of the tannin-silica aerogels

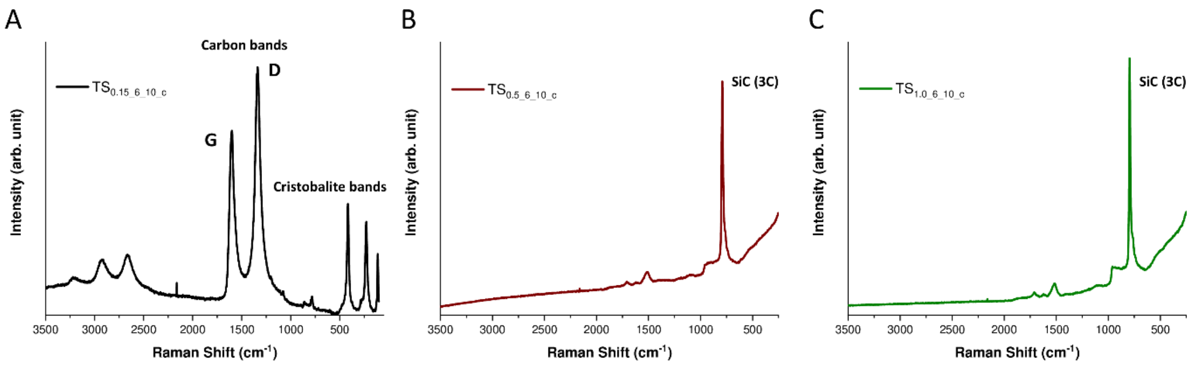

Figure S11 Raman spectra of the carbonized $TS_{0.15\_6\_10\_c}$ (black), $TS_{0.5\_6\_10\_c}$ (red) and $TS_{1.0\_6\_10\_c}$ (green), recorded by utilizing a 532 nm laser wavelength and a power of 4 mW.
